# Does Learning or Instinct Shape Habitat Selection?

**DOI:** 10.1371/journal.pone.0053721

**Published:** 2013-01-16

**Authors:** Scott E. Nielsen, Aaron B. A. Shafer, Mark S. Boyce, Gordon B. Stenhouse

**Affiliations:** 1 Department of Renewable Resources, University of Alberta, Edmonton, Alberta, Canada; 2 Department of Biological Sciences, University of Alberta, Edmonton, Alberta, Canada; 3 Foothills Research Institute and Alberta Sustainable Resource Development, Fish and Wildlife Division, Hinton, Alberta, Canada; University of Tasmania, Australia

## Abstract

Habitat selection is an important behavioural process widely studied for its population-level effects. Models of habitat selection are, however, often fit without a mechanistic consideration. Here, we investigated whether patterns in habitat selection result from instinct or learning for a population of grizzly bears (*Ursus arctos*) in Alberta, Canada. We found that habitat selection and relatedness were positively correlated in female bears during the fall season, with a trend in the spring, but not during any season for males. This suggests that habitat selection is a learned behaviour because males do not participate in parental care: a genetically predetermined behaviour (instinct) would have resulted in habitat selection and relatedness correlations for both sexes. Geographic distance and home range overlap among animals did not alter correlations indicating that dispersal and spatial autocorrelation had little effect on the observed trends. These results suggest that habitat selection in grizzly bears are partly learned from their mothers, which could have implications for the translocation of wildlife to novel environments.

## Introduction

Habitat selection is a behavioural process influencing individual fitness and populations through habitat-specific demographic performance (e.g. [1]–[2]). Patterns of habitat selection form an important basis from which to study evolutionary processes [3]–[4], with those individuals most adept at selecting and using necessary resources (e.g. food, shelter) likely having the highest fitness. While genetics and behaviour are thought to influence habitat selection [5], both hypotheses have largely remained untested in free-ranging wildlife [6].

In altricial and social animals, behavioural patterns could arise from genes or parent-offspring imprinting and/or social learning [7]. To test the effects of genetics and learning, field and laboratory experiments have been proposed [8]–[9], but they are not feasible for most free-ranging animals. Genetic profiling and GPS radiotelemetry now allow estimation of both habitat selection and genetic relatedness for the same individuals in wild populations [10]. Here, we test the competing hypotheses of whether genetics (instinct), or parent-offspring rearing (learning) affect patterns of habitat selection for a population of grizzly bears (*Ursus arctos*) in Alberta, Canada. Grizzly bears are highly mobile omnivores with mother-offspring rearing lasting between 2 and 4 years [11]. In the absence of pedigree information, which would allow the direct examination of parental-offspring relationships, analysis of genetic relatedness within each sex can help tease apart the influence of instinct and learning on behavioural patterns. If maternal rearing were to lead to habitat ‘learning’ or induce a natal habitat preference, we would expect a correlation between patterns of habitat selection and female relatedness, regardless of where individuals reside, and no relationship (or attenuated) among males because most animals would not be siblings - this is analogous to a matrilineal inheritance of behaviour. Because males do not contribute to offspring rearing, a correlation of habitat selection and genetic relatedness in males and females would support a genetically predetermined behaviour of habitat selection.

## Materials and Methods

### Ethics statement

Captures were permitted by, and conducted by the Foothills Research Institute's Grizzly Bear Program as part of long term and ongoing ecological studies in Alberta. All captures were approved by the University of Alberta's Animal Care Committee and are in accordance with the Canadian Council on Animal Care guidelines for handling of wildlife.

### Grizzly bear habitat-use data

**Figure 1:**
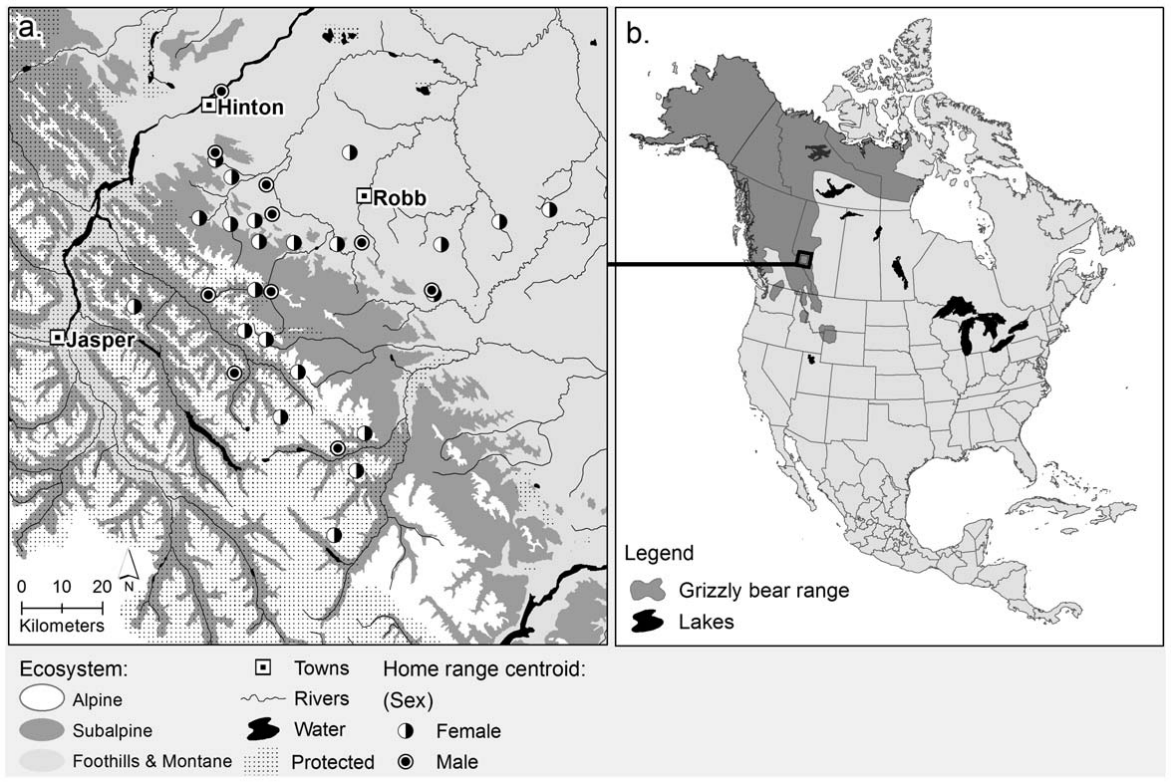
Locations of grizzly bears (*Ursus arctos*) based on home range centroids used to assess the correlation between genetic relatedness and habitat selection in west-central Alberta, Canada (a) and the location of the study area relative to the current range in North America (b).

From 1999 to 2002, we captured 32 adult (>4 years of age) and sub-adult (3–4 years of age) grizzly bears in west-central Alberta, Canada (53°15′N, 118°30′W – Figure 1) using aerial darting and leg snaring [12]. Bears were fitted with either a Televilt GPS-Simplex or an ATS (Advanced Telemetry Systems) GPS radiocollar and programmed to acquire locations at 1-hr and 4-hr intervals. Animal locations were stratified into 3 seasons to account for intra-annual variation in habitat use (Table 1; [13]–[14]). Using a minimum sampling rule of 50 animal locations per season-bear combination, we assessed individual-level habitat selection for 11 land cover classes (Table 2).

**Table 1:** Defined seasons used for assessing habitat use by grizzly bears in west-central Alberta, Canada.

Season	Start date	End date	Characteristic foraging items
Hypophagia (Season 1)	1-May	15-Jun	roots from *Hedysarum* spp., carrion and young ungulate calves
Early hyperphagia (Season 2)	16-Jun	15-Aug	ants (myrmecophagy), *Heracleum lanatum*, graminoids, sedges, and *Equisetum arvense*
Late hyperphagia (Season 3)	16-Aug	15-Oct	fruit (frugivory) of *Vaccinium* spp. and *Shepherdia canadensis*, roots from *Hedysarum* spp.

**Table 2:** Landcover/landuse classes used to represent grizzly bear habitats for assessing habitat use.

Landcover or	Percent	Remote sensing classes
landuse class	composition	
closed conifer forest	37.2	closed coniferous forest
open conifer forest	2.7	open coniferous forest
deciduous forest	3.4	closed & open deciduous forests
mixed forest	7.9	mixed forest
alpine/herbaceous	4.4	alpine/sub-alpine >1800 m & herbaceous <1800 m
open bog/shrub	6.3	open bog & shrub <1800 m
treed bog	5.4	wetland-treed bog
non-vegetated	17	rock, snow/ice, shadow, & water
anthropogenic	3.9	road/rail line, pipleline, well site, & urban
regenerating forest	7.5	clearcuts and recent burns
riparian	4.3	n.a. (obtained through GIS model)

Composition (%) of habitats within the study area are provided, as well as the original remote sensing class used to define grizzly bear habitats.

First, we calculated the proportion use, *u_i_*, of habitat *i* by comparing the number of observations, *n*, observed in each habitat *i* with the total number of observations across all 11 habitats or,
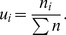
(1)To account for GPS radiotelemetry bias (variance in fix rates), we accounted for the probability of acquiring a GPS acquisition, *p*_(*fix*)_, based on local habitat and terrain covariates [15]. After applying *p*_(*fix*)_ values by habitat class in both GPS radiocollars, we estimated *p*_(*fix*)_ values using zonal statistics in ArcGIS (ESRI, v9.3). Our bias-adjustment of *n_i_* was then defined as,
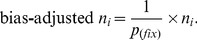
(2)Bias-adjusted values of *n_i_* were fit back into equation 1. We then generated multi-annual 100% minimum convex polygon (MCP) home ranges for each animal to estimate habitat availability [16]. Within individual home ranges, we calculated the proportion availability, *a_i_*, of each habitat class by summing the individual map pixels (30 m cells) within each class and comparing these to the sum of all home range map pixels. We then defined a habitat-selection ratio, *w*_(*x*)_, for each animal following Manly *et al.* [16]:
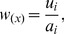
(3)where *u_i_* was defined by equations 1 and 2 and *a_i_* was the proportion availability of habitat *i* based on 100% MCP assessments. Pairwise Pearson correlations for individual animals were estimated based on *w*_(*x*)_ values for each habitat class. The resulting matrix was labeled *S* for habitat selection. We generated a pairwise correlation matrix of habitat availability (*A*) for the same attributes. This was used to account for functional responses in habitat selection where we may expect habitat selection to vary based on the availability of that habitat [17] and to control for correlation in habitat selection among adjacent animals whose home ranges overlap or encompass similar habitats.

### Estimates of genetic relatedness

Root hairs were collected from captured grizzly bears. DNA was isolated and 15 microsatellite loci were amplified according to Proctor *et al.* [18]. Deviations from Hardy–Weinberg equilibrium and linkage disequilibrium were assessed in GENEPOP 4.0 [19]. Genetic relatedness between individuals was estimated using the software SPAGeDi 1.2 [20] and placed into a matrix referred to as *G*.

### Comparing genetic relatedness and animal habitat selection

We used Mantel tests [21] to evaluate the correlation among matrices of habitat selection (*S*) and genetic relatedness (*G*). Because habitat selection can vary as a function of habitat availability [17], we controlled for habitat availability (*A*) using a partial Mantel test; this is denoted by|*A* in the models. We also controlled for home range overlap (*HR*) and geographic distance between home range centroids (*D*), with *D* being the Euclidian distance between individual home range centroids. For the home range overlap, we used each individual's 100% MCP and calculated two-dimensional overlap among pairs of animal home ranges using the geometric mean of the ratio of overlap area to total home range size of each bear [22]. We separated pairwise matrices by gender to test our competing hypotheses and avoid gender-based behavioural differences [23]. This was done because an analysis combining the sexes could elucidate a genetic contribution, but would produce equivocal results if learning were the main driver, thus evaluation of each sex is needed to test the learning versus instinct hypothesis. We applied a global Bonferroni correction to each season correcting for sex comparisons (α = 0.025). All Mantel tests were conducted in R 2.15.0 (http://www.r-project.org/) using the Ecodist package [24] with significance assessed from 10,000 permutations.

## Results

During the 4-year study period, 31,849 locations from 32 animals (10 males, 22 females) were acquired over an area of 9,752-km^2^. Age at time of capture is provided for each individual in Table S1. Seven of the 11 habitats were used similarly among seasons including, alpine/herbaceous, deciduous forest, mixed forest, non-vegetated, open conifer forest, riparian, and treed-bog (Table S1). All microsatellite markers were in Hardy-Weinberg and linkage equilibrium and are available from the Dryad Data Repository (doi:10.5061/dryad.76ks0). The overall observed heterozygosity was 0.65 and each locus averaged 6.3 alleles. The Queller and Goodnight relationship coefficient [25] explained the most variation and was used as the genetic relatedness matrix (*G*). No relationship between genetic relatedness and patterns of habitat selection were observed in males during any season, while there was an overall positive relationship between relatedness and habitat selection in females (Table 3). Notably, female bears in spring (season 1) and late-summer/autumn (season 3) showed a positive relationship between genetic relatedness and habitat selection patterns (*r*_(*G*∼*S*|*A*)_ = 0.24 and 0.11, respectively) with the late-summer autumn period being relatively robust (*p* = 0.02; the spring *p* value was 0.16). When all seasons were combined, a positive correlation between genetic relatedness and shared habitat selection patterns were observed for female grizzly bears (*r*_(*G*∼*S*|*A*)_ = 0.19, *p* = 0.02). In contrast, no relationship was observed between habitat selection and relatedness among male grizzly bears (*r*_(*G*∼*S*|*A*)_ = −0.04, *p* = 0.43). Neither geographic distance nor home range overlap had a tangible effect on the correlations suggesting that distances and shared home range space between animals did not affect the patterns (Table 3).

**Table 3:** Partial Mantel tests showing the correlation between genetic relatedness (*G*) and habitat selection (*S*), when habitat availability (*A*) is controlled for, in grizzly bears from west-central Alberta.

	Female		Male	
	Mantel *r*	*P* value	Mantel *r*	*P* value
Season 1				
*G*∼*S*|*A*	0.11	0.16	−0.10	0.73
*G*∼*S*|*A*+*D*	0.10	0.18	−0.19	0.86
*G*∼*S*|*A*+*HR*	0.09	0.21	−0.11	0.76
*G*∼*S*|*A*+*HR*+*D*	0.08	0.21	−0.12	0.73
Season 2				
*G*∼*S*|*A*	0.01	0.43	−0.22	0.91
*G*∼*S*|*A*+*D*	0.04	0.22	−0.22	0.90
*G*∼*S*|*A*+*HR*	0.02	0.41	−0.20	0.88
*G*∼*S*|*A*+*HR*+*D*	0.04	0.32	−0.20	0.87
Season 3				
*G*∼*S*|*A*	0.25	0.02	0.00	0.62
*G*∼*S*|*A*+*D*	0.22	0.02	−0.02	0.63
*G*∼*S*|*A*+*HR*	0.23	0.02	0.00	0.66
*G*∼*S*|*A*+*HR*+*D*	0.23	0.03	−0.18	0.54

Distance (*D*) and home range overlap (*HR*) are controlled for in additional models. Correlations are shown by sex and broken into three seasons according to Table 1. Significance should be assessed with a global Bonferroni correction making α = 0.025.

## Discussion

Habitat selection varied among grizzly bears supporting previous studies that showed appreciable individual-level variation in grizzly bear behaviour [13], [26]–[27]. This variation was in part explained by genetic relatedness, because related female grizzly bears were more likely to select similar habitats. This relationship was most pronounced in late summer-autumn (to a lesser extent in spring), but was not observed during the middle of summer. In contrast to females, related male animals did not select similar habitats in any season. These results lend support for the ‘habitat-learning’ hypothesis, where maternal parent-offspring rearing transfers knowledge of habitat selection strategies from parent to offspring. Natal habitat preference induction has been observed across taxa [28], but to our knowledge, we are among the first to quantitatively test the genetic versus learning hypotheses of habitat selection *in situ*.

The overall effect in females should be interpreted with caution since it is affected by seasonal differences that suggest specific habitat selection strategies may be more important for some resources and individuals. A closer examination of seasonal food sources helps with understanding the intra-annual variations in habitat selection among related females. During spring (season 1) bears generally used the same riparian and alpine habitats for root digging along with fragmented forests for ungulate calves [27], [29]. In contrast, during late summer/early autumn (season 3) bears relied almost entirely on fruit from distinct patches of fruit-bearing dwarf (*Vaccinium* spp.) and tall shrubs (*Shepherdia canadensis*) [27], [29]. Given the importance of fruit to interior populations of bears that lack access to concentrated resources such as salmon [30], it would seem advantageous to learn which habitat conditions are most suitable for fruit, and depending on the area, which species of fruit to specialize in. For concentrated and nutritionally important resources, individual animals are known to have site fidelity [31]. Fidelity to specific sites and habitats may afford their young the opportunity to learn which habitat to use later in life. In contrast to the fruiting season, during summer months herbaceous food resources are more diverse [27], spatially ubiquitous [29] and of lower quality [32]. We therefore suggest that grizzly bear offspring have fewer cues to learn about specific sites/resources from which to base future habitat selection or specialize on. Indeed when food resources are of generally low quality and spatially ubiquitous, fidelity to specific sites and habitats is low [33].

A large portion of variance still remained unexplained indicating that other factors are at play including, among others, intraspecific competition and inter-annual variability in resources. One mechanism we considered was dispersal, because philopatry is female-biased in grizzly bears [11]. If female offspring do not disperse, they could select similar habitats to their mother due simply to spatial adjacency and habitat availability. However, when distance and home range overlap were considered, our results did not change; any potential mother-daughter pairings are not then likely to be skewing our results further supporting the general phenomenon of habitat learning. Given that grizzly bears reside in highly variable environments, are long-lived, and exhibit considerable ecological plasticity, learning strategies as exhibited here should be adaptive since the same response would not always be optimal [34]. We might expect that individuals deviating from the mean (population) pattern of habitat selection would have negative fitness consequences [35]. Assessing the adaptive significance of variability in habitat selection strategies among animals is therefore important to understanding relationships between habitat selection and animal fitness [36].

While we have shown – in part – a pattern consistent with a learned-basis of habitat selection for grizzly bears, additional studies are required to substantiate these trends. In particular, it would be valuable to examine how patterns of habitat selection vary by age and sex classes across multiple populations of grizzly bears where food resources, habitats, and inter-annual variation differs. Even if learned, a genetic signature in habitat selection would be expected to accrue over time if the same behaviour is selected across multiple generations, thus transforming learning into instinct [37]. We recommend future studies incorporate larger sample sizes, comparisons among taxa with different offspring rearing strategies and food resource patterns. Genomic and quantitative genetics approaches for identification of inheritance or genes specifically associated with behaviour should also be considered. For managed wildlife species where learning plays an important role in determining habitat selection strategies, it should not be overlooked that translocations of animals to novel environments may prove difficult or inefficient if the animal lacks prior experience. As the mechanisms behind habitat selection become better understood, we can assess how these different strategies and mechanisms affect fitness and population dynamics in free-ranging species.

## Supporting Information

Table S1 Habitat selection ratios, *w*_(*x*)_, average seasonal bias-adjusted proportion use, *u_i_*, and proportion availability, *a_i_*, for 7 temporally invariant habitat classes used to describe individual-level habitat selection of grizzly bears in west-central Alberta. Bear ID's are denoted –F for female, or –M for male. Age refers to age at time of capture.
(DOCX)
