# Supplementary material for: Does Learning or Instinct Shape Habitat Selection?
Source: PLoS One. 2013 Jan 16;8(1):e53721. doi: 10.1371/journal.pone.0053721 (PMC3547072; doi:10.1371/journal.pone.0053721)
Supplement: Table S1 — Habitat selection ratios, w (x), average seasonal bias-adjusted proportion use, ui, and proportion availability, ai, for 7 temporally invariant habitat classes used to describe individual-level habitat selection of grizzly bears in west-central Alberta. Bear ID's are denoted –F for female, or –M for male. Age refers to age at time of capture. (DOCX) [file pone.0053721.s001.docx]

**Table S1**. Habitat selection ratios, *w*_(_*_x_*_)_, average seasonal bias-adjusted proportion use, *u_i_*, and proportion availability, *a_i_*, for 7 temporally invariant habitat classes used to describe individual-level habitat selection of grizzly bears in west-central Alberta. Bear ID’s are denoted –F for female, or –M for male. Age refers to age at time of capture.

| Bear ID | Age | closed conifer forest | | |  | deciduous forest | | |  | mixed forest | | |  | non-vegetated | | |  | open conifer forest | | |  | riparian | | |  | treed-bog | | |
| --- | --- | --- | --- | --- | --- | --- | --- | --- | --- | --- | --- | --- | --- | --- | --- | --- | --- | --- | --- | --- | --- | --- | --- | --- | --- | --- | --- | --- |
| and sex |  | *u_i_* | *a_i_* | *w*_(_*_x_*_)_ |  | *u_i_* | *a_i_* | *w*_(_*_x_*_)_ |  | *u_i_* | *a_i_* | *w*_(_*_x_*_)_ |  | *u_i_* | *a_i_* | *w*_(_*_x_*_)_ |  | *u_i_* | *a_i_* | *w*_(_*_x_*_)_ |  | *u_i_* | *a_i_* | *w*_(_*_x_*_)_ |  | *u_i_* | *a_i_* | *w*_(_*_x_*_)_ |
| GB01-M | >4 | 0.244 | 0.098 | 2.495 |  | 0.031 | 0.005 | 6.777 |  | 0.001 | 0.005 | 0.191 |  | 0.235 | 0.616 | 0.382 |  | 0.126 | 0.045 | 2.780 |  | 0.097 | 0.015 | 6.642 |  | 0.059 | 0.013 | 4.615 |
| GB02-F | 18 | 0.288 | 0.096 | 2.989 |  | 0.008 | 0.004 | 1.857 |  | 0.002 | 0.003 | 0.552 |  | 0.387 | 0.714 | 0.542 |  | 0.104 | 0.040 | 2.590 |  | 0.037 | 0.017 | 2.121 |  | 0.034 | 0.014 | 2.390 |
| GB03-F | 5 | 0.356 | 0.122 | 2.919 |  | 0.011 | 0.006 | 1.850 |  | 0.005 | 0.003 | 1.714 |  | 0.225 | 0.509 | 0.442 |  | 0.134 | 0.090 | 1.484 |  | 0.014 | 0.015 | 0.966 |  | 0.009 | 0.011 | 0.832 |
| GB04-F | 5 | 0.462 | 0.192 | 2.414 |  | 0.030 | 0.023 | 1.306 |  | 0.006 | 0.011 | 0.509 |  | 0.213 | 0.402 | 0.530 |  | 0.127 | 0.090 | 1.408 |  | 0.01 | 0.009 | 1.022 |  | 0.029 | 0.021 | 1.373 |
| GB05-M | >4 | 0.046 | 0.088 | 0.520 |  | 0.054 | 0.020 | 2.663 |  | 0.102 | 0.048 | 2.124 |  | 0.059 | 0.245 | 0.240 |  | 0.070 | 0.050 | 1.396 |  | 0.065 | 0.022 | 2.960 |  | 0.031 | 0.009 | 3.576 |
| GB06-M | 16 | 0.373 | 0.146 | 2.554 |  | 0.024 | 0.007 | 3.350 |  | 0.004 | 0.004 | 0.919 |  | 0.171 | 0.584 | 0.292 |  | 0.124 | 0.050 | 2.455 |  | 0.026 | 0.007 | 3.708 |  | 0.024 | 0.005 | 4.710 |
| GB07-F | 3 | 0.001 | 0.005 | 0.208 |  | 0.036 | 0.034 | 1.065 |  | 0.133 | 0.098 | 1.348 |  | 0.048 | 0.013 | 3.856 |  | 0.012 | 0.016 | 0.761 |  | 0.157 | 0.068 | 2.317 |  | 0.036 | 0.018 | 2.069 |
| GB08-M | 14 | 0.253 | 0.101 | 2.490 |  | 0.045 | 0.020 | 2.284 |  | 0.019 | 0.031 | 0.631 |  | 0.086 | 0.266 | 0.324 |  | 0.149 | 0.072 | 2.055 |  | 0.026 | 0.019 | 1.343 |  | 0.052 | 0.015 | 3.480 |
| GB10-F | 13 | 0.442 | 0.138 | 3.209 |  | 0.007 | 0.004 | 1.868 |  | 0.001 | 0.001 | 0.786 |  | 0.222 | 0.624 | 0.357 |  | 0.100 | 0.046 | 2.202 |  | 0.041 | 0.012 | 3.266 |  | 0.016 | 0.004 | 3.780 |
| GB11-F | 6 | 0.010 | 0.007 | 1.429 |  | 0.010 | 0.019 | 0.523 |  | 0.079 | 0.070 | 1.141 |  | 0.014 | 0.030 | 0.447 |  | 0.010 | 0.007 | 1.429 |  | 0.191 | 0.065 | 2.932 |  | 0.026 | 0.022 | 1.143 |
| GB12-F | 5 | 0.007 | 0.003 | 2.281 |  | 0.047 | 0.048 | 0.990 |  | 0.219 | 0.141 | 1.552 |  | 0.035 | 0.018 | 1.934 |  | 0.007 | 0.008 | 0.852 |  | 0.098 | 0.070 | 1.395 |  | 0.061 | 0.066 | 0.917 |
| GB13-F | 4 | 0.020 | 0.004 | 5.154 |  | 0.061 | 0.028 | 2.195 |  | 0.172 | 0.102 | 1.694 |  | 0.041 | 0.021 | 1.929 |  | 0.001 | 0.005 | 0.217 |  | 0.061 | 0.054 | 1.122 |  | 0.207 | 0.099 | 2.085 |
| GB14-M | 9 | 0.029 | 0.021 | 1.359 |  | 0.246 | 0.039 | 6.359 |  | 0.114 | 0.101 | 1.124 |  | 0.033 | 0.042 | 0.782 |  | 0.036 | 0.024 | 1.523 |  | 0.046 | 0.051 | 0.897 |  | 0.024 | 0.022 | 1.092 |
| GB16-F | 5 | 0.502 | 0.173 | 2.899 |  | 0.038 | 0.011 | 3.600 |  | 0.009 | 0.006 | 1.393 |  | 0.172 | 0.490 | 0.351 |  | 0.096 | 0.089 | 1.073 |  | 0.006 | 0.008 | 0.711 |  | 0.042 | 0.012 | 3.609 |
| GB17-M | 7 | 0.028 | 0.022 | 1.286 |  | 0.015 | 0.027 | 0.564 |  | 0.074 | 0.105 | 0.702 |  | 0.033 | 0.045 | 0.717 |  | 0.024 | 0.023 | 1.024 |  | 0.076 | 0.051 | 1.479 |  | 0.016 | 0.013 | 1.284 |
| GB20-F | 4 | 0.041 | 0.027 | 1.502 |  | 0.023 | 0.019 | 1.229 |  | 0.086 | 0.110 | 0.778 |  | 0.019 | 0.036 | 0.515 |  | 0.021 | 0.019 | 1.093 |  | 0.081 | 0.044 | 1.827 |  | 0.011 | 0.009 | 1.213 |
| GB23-F | 11 | 0.044 | 0.032 | 1.368 |  | 0.080 | 0.035 | 2.268 |  | 0.056 | 0.078 | 0.716 |  | 0.038 | 0.072 | 0.531 |  | 0.151 | 0.038 | 3.971 |  | 0.017 | 0.051 | 0.333 |  | 0.040 | 0.017 | 2.283 |
| GB24-M | 6 | 0.003 | 0.013 | 0.274 |  | 0.029 | 0.030 | 0.973 |  | 0.111 | 0.139 | 0.800 |  | 0.030 | 0.021 | 1.412 |  | 0.012 | 0.016 | 0.749 |  | 0.094 | 0.060 | 1.578 |  | 0.020 | 0.019 | 1.047 |
| GB26-F | 3 | 0.035 | 0.004 | 8.775 |  | 0.068 | 0.028 | 2.384 |  | 0.048 | 0.095 | 0.504 |  | 0.118 | 0.019 | 6.114 |  | 0.006 | 0.004 | 1.341 |  | 0.18 | 0.062 | 2.913 |  | 0.038 | 0.101 | 0.370 |
| GB27-F | 11 | 0.030 | 0.006 | 4.825 |  | 0.080 | 0.034 | 2.386 |  | 0.085 | 0.099 | 0.856 |  | 0.053 | 0.021 | 2.491 |  | 0.050 | 0.013 | 3.937 |  | 0.051 | 0.055 | 0.912 |  | 0.068 | 0.069 | 0.988 |
| GB28-F | 6 | 0.362 | 0.095 | 3.827 |  | 0.012 | 0.007 | 1.864 |  | 0.003 | 0.007 | 0.444 |  | 0.283 | 0.416 | 0.681 |  | 0.131 | 0.069 | 1.917 |  | 0.033 | 0.022 | 1.507 |  | 0.020 | 0.024 | 0.831 |
| GB33-M | 3 | 0.020 | 0.019 | 1.073 |  | 0.121 | 0.039 | 3.145 |  | 0.098 | 0.088 | 1.105 |  | 0.049 | 0.092 | 0.537 |  | 0.018 | 0.020 | 0.908 |  | 0.103 | 0.052 | 1.990 |  | 0.056 | 0.069 | 0.807 |
| GB34-F | 21 | 0.340 | 0.111 | 3.072 |  | 0.011 | 0.005 | 2.140 |  | 0.001 | 0.005 | 0.204 |  | 0.253 | 0.595 | 0.425 |  | 0.043 | 0.050 | 0.855 |  | 0.108 | 0.017 | 6.195 |  | 0.015 | 0.016 | 0.956 |
| GB35-F | 3 | 0.629 | 0.196 | 3.213 |  | 0.007 | 0.012 | 0.542 |  | 0.001 | 0.008 | 0.128 |  | 0.222 | 0.519 | 0.427 |  | 0.064 | 0.058 | 1.107 |  | 0.004 | 0.007 | 0.486 |  | 0.008 | 0.014 | 0.580 |
| GB36-F | 3 | 0.057 | 0.023 | 2.465 |  | 0.041 | 0.024 | 1.710 |  | 0.106 | 0.129 | 0.827 |  | 0.039 | 0.039 | 1.010 |  | 0.021 | 0.020 | 1.051 |  | 0.072 | 0.044 | 1.638 |  | 0.013 | 0.009 | 1.438 |
| GB37-F | 3 | 0.107 | 0.063 | 1.695 |  | 0.022 | 0.028 | 0.762 |  | 0.039 | 0.062 | 0.627 |  | 0.072 | 0.151 | 0.478 |  | 0.060 | 0.045 | 1.322 |  | 0.041 | 0.026 | 1.593 |  | 0.016 | 0.010 | 1.698 |
| GB38-F | 15 | 0.155 | 0.086 | 1.795 |  | 0.043 | 0.023 | 1.903 |  | 0.041 | 0.030 | 1.372 |  | 0.062 | 0.117 | 0.526 |  | 0.079 | 0.052 | 1.516 |  | 0.004 | 0.017 | 0.254 |  | 0.006 | 0.006 | 1.164 |
| GB40-F | 4 | 0.084 | 0.047 | 1.778 |  | 0.052 | 0.024 | 2.128 |  | 0.047 | 0.072 | 0.654 |  | 0.111 | 0.118 | 0.946 |  | 0.138 | 0.035 | 3.921 |  | 0.037 | 0.041 | 0.908 |  | 0.031 | 0.011 | 2.702 |
| GB42-F | 18 | 0.200 | 0.103 | 1.944 |  | 0.026 | 0.018 | 1.431 |  | 0.016 | 0.025 | 0.627 |  | 0.166 | 0.411 | 0.404 |  | 0.115 | 0.040 | 2.859 |  | 0.012 | 0.002 | 6.833 |  | 0.001 | 0.001 | 1.000 |
| GB50-M | 4 | 0.006 | 0.012 | 0.470 |  | 0.044 | 0.022 | 1.965 |  | 0.135 | 0.149 | 0.908 |  | 0.006 | 0.017 | 0.332 |  | 0.011 | 0.010 | 1.055 |  | 0.093 | 0.048 | 1.922 |  | 0.011 | 0.009 | 1.137 |
| GB54-M | 4 | 0.009 | 0.022 | 0.394 |  | 0.026 | 0.024 | 1.079 |  | 0.071 | 0.089 | 0.792 |  | 0.009 | 0.031 | 0.285 |  | 0.009 | 0.024 | 0.366 |  | 0.088 | 0.045 | 1.969 |  | 0.001 | 0.011 | 0.089 |
| GB100-F | 3 | 0.030 | 0.063 | 0.478 |  | 0.055 | 0.035 | 1.576 |  | 0.067 | 0.059 | 1.139 |  | 0.055 | 0.150 | 0.368 |  | 0.070 | 0.049 | 1.421 |  | 0.016 | 0.031 | 0.521 |  | 0.037 | 0.014 | 2.624 |
